# Role of Artificial Intelligence (AI) in Patient Education and Communication in Dentistry

**DOI:** 10.7759/cureus.59799

**Published:** 2024-05-07

**Authors:** Vinayak Thorat, Prajakta Rao, Nilesh Joshi, Prakash Talreja, Anupa R Shetty

**Affiliations:** 1 Department of Periodontology, Bharati Vidyapeeth (Deemed to be University) Dental College and Hospital, Navi Mumbai, IND

**Keywords:** artificial intelligence in dentistry, ai applications in dentistry, dental patient engagement, ai in dentistry, patient education ai, dental ai communication

## Abstract

Effective patient education and communication are integral components of quality dental care, contributing to informed decision-making, treatment compliance, and positive clinical outcomes. However, traditional methods face challenges such as language barriers, anxiety, and information retention issues. Artificial intelligence (AI) presents innovative solutions to enhance patient engagement and communication in dentistry. This review explores the transformative role of AI in redefining patient education and communication strategies, focusing on applications, benefits, challenges, and future directions. A literature search identified articles from 2018 to 2024, encompassing empirical evidence and conceptual frameworks related to AI in dental patient engagement and communication. Key findings reveal AI's potential to offer personalized educational materials, virtual consultations, language translation tools, and virtual reality simulations, improving patient understanding and experience. Despite advancements, concerns about overreliance, accuracy, implementation costs, patient acceptance, privacy, and regulatory compliance persist. Future implications suggest AI's ability to track patient progress, analyze feedback, streamline administrative processes, and provide ongoing support, enhancing oral health outcomes. However, ethical, regulatory, and equity considerations require attention for responsible AI deployment and widespread adoption. Overall, AI holds promise for revolutionizing dental patient education, communication, and care delivery, emphasizing the need for comprehensive strategies to address emerging challenges and maximize benefits.

## Introduction and background

Effective patient education and communication are fundamental aspects of providing quality dental care. Educating patients about their oral health, treatment options, and preventive measures not only empowers them to make informed decisions but also enhances their compliance with treatment plans. Furthermore, clear and open communication between dental professionals and patients fosters trust, improves patient satisfaction, and ultimately contributes to better clinical outcomes. Research has shown that patient education and communication are critical factors in promoting oral health literacy and reducing dental anxiety and fear [1]. Traditional methods of explaining dental treatment to patients face challenges such as language barriers, a lack of visual aids, and time constraints. Additionally, patients may experience anxiety and have difficulty retaining information.

Artificial intelligence (AI) offers solutions by providing personalized educational materials, virtual consultations, and language translation tools. Virtual reality simulations can reduce fear by familiarizing patients with procedures, while automated reminders and follow-ups reinforce information. Artificial intelligence ensures consistency in information across practices, improving patient understanding and the overall experience. Through these AI-driven solutions, dental professionals can enhance communication, education, and patient satisfaction in treatment explanations. Artificial intelligence encompasses various technologies that enable machines to simulate human intelligence and perform tasks that typically require human cognition, such as learning, problem-solving, and decision-making. In dentistry, AI has emerged as a transformative tool with diverse applications across different domains, including diagnosis, treatment planning, and patient management. Some notable AI applications in dentistry include image analysis for radiographic interpretation, predictive modeling for risk assessment, and virtual simulations for treatment planning [2].

The integration of AI technologies has the potential to revolutionize patient education and communication in dental practice by offering innovative solutions to enhance the delivery of information, facilitate meaningful interactions, and improve patient engagement. This review aims to explore the transformative role of AI in redefining patient education and communication strategies in dentistry, with a focus on its applications, benefits, challenges, and future directions.

## Review

Methodology

A literature search was conducted across electronic databases, including Google, PubMed, Scopus, and Web of Science, to identify relevant articles published from 2018 to 2024. Grey literature sources, such as conference proceedings and organizational websites, were also consulted to ensure comprehensive coverage of the topic. Keywords like "AI in patient education," "AI in dental communication," and "machine learning in dentistry" were used to identify relevant studies and articles.

Articles, reviews, conference papers, and websites were included if they specifically addressed the role of AI in patient education and communication within dental practice settings, provided empirical evidence or conceptual frameworks, and were published in English. Studies outside the specified time frame or not directly related to the application of AI in dental patient engagement and communication were excluded.

The selected sources were systematically evaluated to extract key information, including study methodologies, AI technologies utilized, patient engagement strategies, and outcomes. All co-authors contributed equally to the selection and extraction process to ensure thoroughness and accuracy in data collection. A critical analysis and summary of the existing literature were conducted to provide an overview of the available research and its quality. This involved synthesizing findings and insights from the selected sources to identify trends, challenges, and opportunities in the application of AI in dental patient education and communication.

Ethical considerations and limitations of AI in dentistry were discussed based on current ethical frameworks, regulatory guidelines, and expert opinions in the field. Future implications and conclusions were drawn from the synthesis of existing literature, with an emphasis on potential advancements, challenges, and ethical considerations shaping the future landscape of AI-driven dentistry. 

The review of literature and research revealed a wide range of applications of AI in dentistry, encompassing areas such as diagnostic imaging, treatment planning, administrative tasks, and patient care. However, for the purpose of this review, the focus was specifically on AI applications related to patient education and communication in dental practice.

The results highlighted various AI-driven technologies and platforms designed to enhance patient engagement, comprehension, and satisfaction through personalized content delivery, virtual assistants, teledentistry, immersive technologies (such as virtual reality and augmented reality), and gamification. These AI-enabled solutions address common challenges in patient communication, including language barriers, dental anxiety, and limited oral health literacy, by providing tailored educational materials, interactive experiences, and remote support.

Overall, the results showed the transformative potential of AI in dentistry, particularly in enhancing patient engagement, comprehension, and satisfaction through innovative technological solutions.

Challenges in patient education and communication

Primary obstacles to effective communication encompass constrained time availability, financial incentives favoring treatment-centric approaches over preventive measures, insufficient training in oral health literacy (OHL), the scarcity of patient education materials in plain language, and the challenge of engaging patients with limited OHL proficiency [3]. These obstacles are detailed below.

Time Constraints During Appointments

Limited appointment times restrict the dentist's ability to thoroughly explain procedures and address patient concerns comprehensively. Longer appointment times are associated with improved patient satisfaction and communication.

Financial Incentives

Treatment-centric approaches driven by financial incentives can overshadow preventive measures. This can impact the focus on patient education and communication about oral health maintenance.

Oral Health Literacy

Insufficient training in OHL poses a challenge, as patients may struggle to understand complex dental terminology. This can impede effective communication and decision-making regarding treatment options.

Language Barriers

Language differences can hinder effective communication, affecting patient satisfaction, treatment adherence, and overall health outcomes. Plain-language materials are often scarce, exacerbating this challenge.

Limited Visual Aids

Visual aids are crucial for enhancing patients' understanding of dental procedures. However, their absence or inadequacy can hinder the comprehension and recall of important information.

Dental Anxiety and Fear

Dental anxiety is common among patients and can hinder effective communication during treatment explanations. Addressing emotional needs and providing supportive communication can help alleviate anxiety and enhance patient satisfaction.

Artificial intelligence and its role in dentistry

Artificial intelligence is a branch of computer science dedicated to creating systems and machines that can perform tasks requiring human-like intelligence. These systems are designed to simulate cognitive functions such as learning, reasoning, problem-solving, perception, and decision-making. The ultimate goal of AI is to develop machines that can mimic human intelligence to a degree where they can understand natural language, recognize patterns, adapt to new situations, and exhibit creativity.

In dentistry, AI technologies primarily encompass machine learning (ML) and natural language processing (NLP). Machine learning algorithms enable computers to analyze large datasets, identify patterns, and make predictions or decisions without explicit programming. In dental applications, ML algorithms are used for tasks such as image analysis, diagnosis, treatment planning, and predictive modeling. Natural language processing focuses on enabling computers to understand and interpret human language. Natural language processing algorithms can analyze text data from electronic health records, patient communication platforms, and online resources to extract relevant information, generate responses, and facilitate communication between dental professionals and patients [4].

Role of AI in patient education and communication

Personalized Content Delivery

Personalized content delivery in AI involves tailoring information and resources to meet the specific needs and preferences of individual users through data analysis and ML algorithms. An AI algorithm is a set of rules or instructions that enables AI systems to perform specific tasks or solve particular problems. It processes input data through mathematical or logical operations to produce output or decisions. Common types include supervised learning, unsupervised learning, reinforcement learning, deep learning, and NLP algorithms. These algorithms are essential for tasks like classification, clustering, and language processing, enabling AI systems to perform various functions across different domains. Artificial intelligence algorithms can analyze various types of patient data, including medical history, demographic information, treatment records, and even patient interactions with educational materials. By processing these data, AI can identify patterns, preferences, and specific needs of individual patients [5]. If a patient expresses a preference for visual learning, AI can prioritize the delivery of educational videos or interactive simulations over textual information. Artificial intelligence algorithms can identify common concerns among patients based on the analysis of patient data and interactions. For instance, if many patients exhibit anxiety or fear related to dental procedures, AI can tailor educational content to address these concerns by providing information about pain management techniques, relaxation exercises, or testimonials from other patients who have successfully overcome dental anxiety. By addressing common concerns proactively, personalized education helps alleviate patient anxiety and builds trust in the dental practice. 

Artificial intelligence-powered platforms can generate detailed explanations of dental procedures in a format that aligns with patients' comprehension levels and learning styles. Using NLP capabilities, AI can convert complex dental terminology into layman's terms, making it easier for patients to understand. Additionally, AI can utilize visual aids such as 3D animations or virtual reality simulations to illustrate procedures step-by-step, enhancing patient comprehension and reducing uncertainty or apprehension.

Artificial intelligence algorithms can analyze patient data to identify opportunities for promoting specific oral health habits tailored to each individual. Artificial intelligence can also provide ongoing support and reminders to reinforce positive behaviors, such as sending notifications to encourage regular dental check-ups or tracking progress toward oral health goals. By delivering personalized educational content that resonates with patients' needs, preferences, and learning styles, AI enhances engagement and comprehension. Patients are more likely to pay attention to information that is relevant and tailored to their specific circumstances, leading to better retention and understanding of key concepts.

Virtual Assistants and Chatbots

Artificial intelligence-driven virtual assistants and chatbots are software applications designed to simulate human conversation using AI techniques. Virtual assistants and chatbots are programmed to understand and interpret natural language input from patients. They can process text-based queries and commands, allowing patients to communicate with them in a conversational manner. Chatbots are software applications powered by AI that simulate human conversation through text or voice interactions. They use NLP algorithms to understand and respond to user queries or commands, providing information, assistance, or performing tasks based on predefined rules or learned patterns. Chatbots are commonly used in various applications, including customer service, online support, virtual assistants, and more recently, in healthcare for patient communication and engagement.

Awrel's virtual assistant, integrated into dental websites, offers customizable AI interactions via text or voice [6]. It guides new patients by providing personal information, assessing symptoms, suggesting self-care regimens, and scheduling in-person appointments if needed. All data shared are Health Insurance Portability and Accountability Act (HIPAA)-compliant and are seamlessly transferred to patient records through Awrel's software. Built on previous texting and voice assistant apps, this project enhances patient engagement and practice efficiency. Virtual assistants and chatbots are equipped with knowledge bases or databases containing a wide range of information related to dental procedures, treatments, oral health, and post-treatment care. When patients ask questions, these AI-driven systems use NLP algorithms to analyze the queries and retrieve relevant information from their knowledge bases. They can provide accurate and timely answers to common questions about dental conditions, treatment options, appointment scheduling, insurance coverage, and more [7].

Patients often have questions or concerns about specific dental treatments, procedures, or interventions. Virtual assistants and chatbots can deliver detailed explanations and information about various dental treatments, including their purposes, processes, benefits, and potential risks. It can offer personalized guidance, recommendations, and reminders to patients regarding proper oral hygiene practices, such as brushing techniques, flossing, dietary habits, and the use of dental products [8]. Additionally, they can provide instructions and tips for post-treatment care to help patients recover smoothly and prevent complications.

After dental procedures, chatbots can check in with patients to assess their recovery progress. They ask about pain levels, medication adherence, and any unexpected symptoms. This proactive approach ensures timely intervention if complications arise. Dental anxiety is common, and chatbots can play a role in easing patients' fears. By providing friendly interactions, answering questions, and explaining procedures, chatbots create a more comfortable experience [9].

Role of AI-Enabled Software and Smartwatches

Image analysis software tools in dentistry leverage advanced AI and machine learning algorithms to assist dental professionals in interpreting radiographic images and diagnosing various dental conditions. These software applications are designed to analyze dental radiographs, including intraoral and extraoral X-rays, panoramic radiographs, and cone-beam computed tomography (CBCT) scans, with the goal of improving diagnostic accuracy, efficiency, and workflow optimization [10].

Dental professionals can use the visual output generated by image analysis software to illustrate and explain dental conditions to patients. By showing patients their radiographs and pointing out abnormalities detected by the software, dentists can enhance patients' understanding of their oral health status and the need for specific treatments. By visualizing their dental radiographs and gaining insights into their oral health conditions, patients develop a greater awareness of the importance of preventive care and regular dental check-ups. The visual representation of dental issues can motivate patients to adopt healthier oral hygiene practices and seek timely treatment when necessary.

Pearl, a dental AI platform, has published results on patients' trust in dental providers and their perceptions of technology in the dental field. The Dental Patient Trust & Technology Survey report, based on responses from 597 U.S. dental patients, revealed that patients overwhelmingly support the use of advanced technologies, particularly AI, in dental diagnosis [11].

Artificial intelligence-driven communication platforms also streamline appointment scheduling processes by offering automated scheduling, reminders, and follow-up messages. These systems reduce no-show rates, improve appointment adherence, and enhance communication between patients and dental practices. Patient Prism LLC [12] is a management software that uses NLP technology and human call coaching validation to connect patients directly with dental practices. By tracking and analyzing new patient calls, it helps practices identify and schedule high-value patients, receive alerts, and receive expert call coaching to capitalize on missed opportunities. It can also train team members and gain real-time intelligence to enhance staffing, patient experience, and practice marketing. With advanced NLP technology, it interprets dental-related phone conversations with up to 95% accuracy.

Artificial intelligence technology, when effectively implemented, simulates human behavior and capabilities, making it appealing for various applications. One such application is AI-based wristwatches, which are gaining popularity in the consumer market, especially for health monitoring. Users, primarily aged between 19 and 30, both male and female, are adopting these smart watches for health alerts [13]. These gadgets not only monitor health but also provide valuable health information, influencing health decisions and promoting health education. Smartwatches can send reminders for brushing and flossing through gentle vibrations. They integrate dental health modules into health apps, allowing users to log routines, set reminders, and receive feedback.

Role of AI-Enabled Teledentistry

Teledentistry merges telecommunications with dentistry, enabling the exchange of clinical data and images over remote distances for dental consultation and treatment planning. It enhances access to oral healthcare, optimizes its delivery, and reduces costs. Moreover, teledentistry holds promise for mitigating oral healthcare disparities between rural and urban areas. Artificial intelligence-enhanced teledentistry can transform dental care from reactive to proactive, personalized prevention.

Teledentistry platforms like Dentulu, Toothpic, Rhinogram, Review Tools, Smile Snap, Teledentix, TeleDent, Carestack, and Dental Monitoring ensure HIPAA compliance and end-to-end encryption for patient data. Dedicated versions of mainstream platforms, like Zoom for Healthcare and G Suite Meet, can also be configured for HIPAA compliance. However, caution is advised with platforms like WhatsApp, Apple FaceTime, and Facebook Messenger, as they aren't HIPAA-compliant [14]. These teledentistry platforms utilize AI for tasks such as diagnosis, sharing virtual records, monitoring progress, providing alerts for complications, and promoting dental health awareness. Remote oral screening with AI can revolutionize preventive care by predicting oral cancers and diagnosing conditions like squamous cell carcinoma through intraoral photographs or smartphone-based probes, even in high-risk populations [15].

Studies conducted by Palmer et al. and Estai et al. highlight the positive reception of teledentistry among dental professionals, emphasizing its ability to substantially enhance patient care and facilitate consultations among specialists [16]. This reception is particularly significant in rural and urban healthcare settings, where teledentistry can reduce healthcare disparities and enable individuals in remote areas to access specialist care.

The growing prevalence of dental apps and the expansion of mobile health (M-health) further enrich the potential of teledentistry in patient education. Incorporating teledentistry into dental practice also extends to digital workflows and digital communication tools, offering dentists opportunities to streamline processes and enhance patient interactions [17]. Despite concerns about infrastructure inadequacy, dental professionals express willingness to allocate time for enhancing their knowledge of teledentistry tools, highlighting the importance of continuous learning and infrastructure development in maximizing the benefits of teledentistry.

The adoption of teledentistry is not limited by age or gender, as evidenced by consistent usage patterns across different demographics. Moreover, teledentistry tools hold promise for improving oral health literacy and patient outcomes, as they offer structured education programs and continuous monitoring of patients' oral health status. Establishing professional oversight and ensuring the suitability of recommended applications are essential steps in leveraging teledentistry for patient education effectively.

Furthermore, digital workflow tools such as 3D planning, printing, and surgical templates are recognized for their potential to enhance dental practice, with dentists expressing a desire to incorporate these technologies into their daily routines [18]. These advancements align with the preferences of dental specialties such as radiology and prosthodontics, where digitalization offers benefits in areas like impression-taking and laboratory work phases.

Utilizing gamified online role-play as a learning strategy for teledentistry can significantly impact education by simulating real-life scenarios. This pedagogical approach allows dental learners to gain practical experience in teledentistry within a safe and controlled environment before engaging with actual patients [19].

Role of AI-enabled Virtual Reality (VR) and Augmented Reality (AR)

Virtual reality and augmented reality technologies are revolutionizing the field of dentistry by offering patients immersive and interactive experiences that enhance their understanding of and engagement with dental care. Through VR headsets or AR devices, patients can step into a virtual dental clinic environment, where they can observe and even participate in simulated dental procedures such as extractions, implants, or root canals [20]. This not only provides patients with a visual representation of what to expect during their actual dental visit but also helps alleviate the anxiety and fear often associated with dental procedures. For example, patients considering orthodontic treatment can use AR applications to see how braces or aligners will affect their smile before committing to treatment. This level of visualization empowers patients to make informed decisions about their oral health care and increases their confidence in the treatment process. 

In addition to visualizing dental procedures, VR and AR applications allow patients to explore the intricacies of dental anatomy in a hands-on and interactive manner. Using 3D models and animations, patients can virtually dissect teeth, gums, and other oral structures, gaining a deeper understanding of their anatomy and function. This immersive educational experience goes beyond traditional methods of patient education, as it provides a dynamic and engaging way for patients to learn about their oral health [21].

Virtual reality and augmented reality technologies are valuable tools for patient education and oral hygiene instruction. Patients can interact with virtual toothbrushes, dental floss, and mouthwash to learn proper brushing, flossing, and rinsing techniques [22]. These immersive educational experiences not only reinforce good oral hygiene habits but also promote better oral health outcomes by educating patients to take an active role in their dental care regimen.

Moreover, during dental procedures, VR environments can be utilized to manage pain and discomfort by immersing patients in calming and relaxing virtual scenes. This distraction technique helps alleviate anxiety and stress, improves communication, and makes the dental experience more tolerable for patients. Similarly, AR applications can provide interactive distractions, such as games or visualizations, to keep patients, especially children, engaged and relaxed during treatment [23].

Role of AI-Enabled Gamification for Patient Engagement

Artificial intelligence-enabled gamification introduces an innovative approach to patient education and communication, offering dynamic and engaging learning experiences through interactive methods. A study done by Zolfaghari et al. [24] compared a basic oral healthcare app with a gamified version to assess its impact on mothers' knowledge and practices regarding children's oral health. Both versions improved mothers' knowledge and practices, but the gamified application led to a greater reduction in children's plaque index, indicating superior clinical outcomes. Dental gamification utilizes principles from behavioral psychology to motivate patients and engage them in their oral health care.

By offering real-time feedback, personalized experiences, and social elements, gamified systems create an immersive environment that encourages users to actively participate in their dental hygiene routines. Immediate feedback on brushing habits or gum health helps users understand their progress and areas for improvement, reinforcing positive behaviors and prompting adjustments when necessary [25].

Personalization ensures that challenges, goals, and rewards resonate with each individual, making the experience more relevant and engaging. Social dynamics, such as leaderboards and community interactions, foster a sense of camaraderie and competition among users, motivating them to improve their oral hygiene habits [26]. Additionally, incorporating narratives and storytelling into the gamified experience adds depth and emotional connection, making it more enjoyable and memorable.

Ethical Considerations

The utilization of AI in clinical settings holds tremendous potential for enhancing healthcare outcomes, yet it also brings forth ethical considerations that demand our attention. To realize the full benefits of AI in healthcare, it's imperative to address four key ethical issues, which are as follows: (1) obtaining informed consent for data usage; (2) ensuring safety and transparency; (3) evaluating algorithmic fairness and biases; (4) safeguarding data privacy [27].

Firstly, the ethical use of AI in healthcare necessitates addressing issues related to patient autonomy and informed consent. Artificial intelligence systems have the potential to influence medical decision-making, highlighting the importance of ensuring that patients are adequately informed about the role of AI in their care. Patients should have the right to understand and consent to AI-driven interventions, with the option to opt-out if desired.

Secondly, the transparency and interpretability of AI algorithms raise ethical concerns. Many AI models, particularly deep learning neural networks, are often perceived as "black boxes" due to their complex decision-making processes. This lack of interpretability poses challenges for accountability, as healthcare professionals may struggle to understand and justify AI-generated recommendations. Addressing this challenge requires the development of transparent AI systems and the establishment of clear guidelines for accountability.

Thirdly, the issue of bias in AI algorithms is a significant ethical concern. Artificial intelligence models trained on biased or incomplete datasets may perpetuate or exacerbate existing healthcare disparities. For instance, biases based on race or gender present in historical data may manifest in AI-driven diagnostic or treatment recommendations. Mitigating bias requires ensuring diverse and representative training data, rigorous algorithm testing, and ongoing monitoring to prevent discriminatory outcomes.

Lastly, the ethical implications of AI in healthcare encompass concerns regarding privacy and data security. Artificial intelligence systems heavily rely on sensitive patient data for training algorithms and making accurate predictions. It is imperative to safeguard the privacy and security of these data to uphold patient trust and prevent potential breaches. Implementing robust data governance frameworks, stringent access controls, and anonymization techniques are vital steps to mitigate these risks.

To sum up, the increasing adoption of AI in dentistry is accompanied by ethical concerns that are not adequately addressed. It highlights a significant gap in ethical discourse and a lack of transparency in sharing AI algorithms, which could hinder future research efforts. A more responsible and transparent use of AI in dental applications is recommended.

Limitations of AI 

There is a risk of overreliance on technology, potentially diminishing the human element in dental care. This could impact the quality of patient-provider relationships and the comprehensiveness of care delivered, emphasizing the importance of maintaining a balanced approach. Despite advancements in AI algorithms and image analysis software, concerns persist regarding their accuracy and reliability. This underscores the need for ongoing validation and supervision by dental professionals to mitigate the risk of misdiagnoses or errors.

The integration of AI, VR/AR, and gamification into dental practices may face significant cost and implementation challenges, particularly for smaller practices or those with limited resources. Patient adoption and acceptance of these technologies may vary, highlighting the importance of addressing usability and user experience concerns [28]. The integration of AI and teledentistry in dental care offers promising avenues for enhancing access to services, particularly in underserved communities or regions with limited internet connectivity.

Moreover, the utilization of AI and teledentistry entails handling sensitive patient data, and raising valid concerns about privacy breaches and security vulnerabilities if adequate safeguards are not implemented. Ensuring regulatory compliance, such as maintaining adherence to HIPAA standards for teledentistry platforms, presents complex challenges that require ongoing monitoring and adaptation to evolving regulations [29].

Realizing the advantages of AI-powered health tools requires acknowledging and addressing the factors influencing patients' online behavior, such as their preferences, expectations, and constraints. To leverage AI effectively, patients must possess a sufficient level of health literacy, meaning they understand how to effectively access and use health information.

Using wearable devices like smartwatches for disease detection offers numerous advantages, including passive testing and continuous monitoring. While smartwatches can track vital signs and symptoms, they may not meet medical standards for diagnosis [30]. Trustworthiness is questioned due to opaque algorithms and varying results among different models. Further research is necessary to unlock the full potential of smartwatches in healthcare and develop more advanced clinical AI assistants.

Future Implications

In addition to the previously mentioned implications, AI stands to revolutionize dental patient education and communication in several other ways. Artificial intelligence-driven apps and platforms can track patients' oral health progress over time, offering personalized feedback and recommendations for improvement, thus fostering accountability and motivation. Moreover, AI algorithms can analyze patient feedback to identify trends and areas for improvement in dental services and patient education efforts, leading to better patient experiences and outcomes. Furthermore, AI can streamline administrative processes related to oral health insurance, freeing up time and resources for patient education initiatives. Lastly, AI-powered coaching platforms can provide ongoing support and guidance to patients, helping them achieve their oral health goals through personalized recommendations and encouragement. Through these diverse applications, AI holds immense promise for enhancing dental patient education, communication, and overall oral health outcomes. The future landscape of AI-driven dentistry also entails personalized treatment and prevention strategies, leveraging predictive modeling and analytics to tailor interventions to individual patient needs and risk profiles. However, alongside technological advancements, future considerations must address ethical and regulatory challenges. Establishing robust ethical frameworks and governance mechanisms is crucial for ensuring responsible AI deployment and safeguarding patient rights. Education and training programs will also need to evolve to equip dental professionals with the requisite skills and knowledge to leverage AI effectively. Building patient acceptance and trust through transparent communication and demonstrated clinical effectiveness will be essential for widespread adoption. Additionally, efforts to promote health equity and access should focus on developing inclusive AI-enabled solutions that cater to diverse populations and address disparities in oral health outcomes. To sum up, the future of AI in dentistry holds immense potential for transforming patient care, driving innovation, and advancing oral health globally.

## Conclusions

In conclusion, the integration of AI into dental practice holds immense promise for overcoming challenges in patient interactions and management, ultimately leading to improved clinical outcomes and patient satisfaction. Through personalized content delivery, virtual assistants, image analysis software, teledentistry platforms, and immersive technologies like VR and AR, AI-driven solutions address language barriers, enhance patient comprehension, and alleviate dental anxiety. Artificial intelligence-enabled tools facilitate personalized education, streamline appointment scheduling, and provide remote diagnostic and treatment support, thereby enhancing access to dental care and promoting proactive prevention. However, ethical considerations, including informed consent, transparency, algorithmic fairness, and data privacy, must be prioritized to ensure the responsible and equitable deployment of AI in dentistry. Despite potential limitations and challenges related to cost, implementation, and patient acceptance, the transformative potential of AI in dentistry cannot be understated. By embracing AI technologies while upholding ethical standards and addressing implementation challenges, dental professionals can revolutionize patient engagement and communication, ultimately advancing the delivery of quality dental care. As AI continues to evolve, ongoing research, validation, and adaptation will be essential to harnessing its full potential and optimizing patient outcomes in dental practice.
